# Process of Reliability of the Ventilatory Workload Kinetic Index and Prioritization in the Intrahospital Clinical Setting

**DOI:** 10.3390/medicina60111770

**Published:** 2024-10-29

**Authors:** Loretto Godoy-Abarca, Andrea Muñoz-Vega, Ramón Pinochet Urzúa, Mariano del Sol, Máximo Escobar-Cabello, Jorge Valenzuela Vásquez, Fernando Valenzuela-Aedo, Camila Díaz-Caro, Francisco Javier Soto-Rodríguez, Rodrigo Muñoz-Cofré

**Affiliations:** 1 Servicio de Medicina Física y Rehabilitación, Hospital El Carmen, Maipú, Santiago 9251521, Chile; lorettogodoy@gmail.com (L.G.-A.); andrea.munoz.vega@gmail.com (A.M.-V.); jvalenzuelavasq@gmail.com (J.V.V.); camila.p.diaz.c@gmail.com (C.D.-C.); 2 Servicio de Kinesiología, Hospital Padre Alberto Hurtado, San Ramón 8880465, Chile; rpinochetu@gmail.com; 3 Programa de doctorado en Ciencias Morfológicas, Universidad de La Frontera, Temuco 4811230, Chile; mariano.delsol@ufrontera.cl (M.d.S.); fernando.valenzuela@ufrontera.cl (F.V.-A.); 4 Centro de Excelencia en Estudios Morfológicos y Quirúrgicos, Universidad de La Frontera, Temuco 4811230, Chile; 5 Laboratorio de Función Disfunción Ventilatoria, Departamento de Kinesiología, Universidad Católica del Maule, Talca 3530000, Chile; 6 Facultad de Medicina, Departamento de Ciencias de la Rehabilitación, Universidad de La Frontera, Temuco 4811230, Chile; francisco.soto@ufrontera.cl; 7 Programa de Doctorado en Medicina Clínica y Salud Pública, Universidad de Granada, 18071 Granada, Spain

**Keywords:** physiotherapy care, clinical instrument, reliability

## Abstract

*Background and Objectives*: The Ventilatory Workload Kinetic Index (VWKI) has been proposed as a clinical instrument to evaluate ventilatory balance–imbalance. However, the regulated application of scales that allow an integral evaluation of the object of study and their subsequent reliability evaluation should be continuous. The objective was to determine the reliability of the VWKI between two evaluators and its applicability in the clinical field of physiotherapy. *Materials and Methods*: The methodology was divided into three stages: (i) induction period, (ii) application of the VWKI, and (iii) assessment of reliability. *Results*: The VWKI total score obtained excellent inter-rater reliability (ICC = 0.913, *p* < 0.05). The airway resistance (AR) showed moderate inter-rater reliability (ICC = 0.528, *p* < 0.05), while the use of accessory musculature (UAM) showed poor inter-rater reliability (ICC = 0.483, *p* < 0.05). In the physiotherapy care prioritization system, for both evaluator A (EA) and evaluator B (EB), the total VWKI score was significantly higher in priority 1 (*p* = 0.001; *p* < 0.05, respectively). Regarding the total VWKI score by services, it was observed that both EA and EB rated the VWKI significantly higher in the intensive care unit (ICU) than in medicine and the other services (*p* = 0.001, *p* = 0.0001 and *p* < 0.05, *p* < 0.05, respectively). *Conclusions*: VWKI is highly reliable in the total score. It is also consistent with the system of prioritization of physiotherapy care and the ability to determine the severity of clinical respiratory symptoms.

## 1. Introduction

In the clinical setting, assessing respiratory impairment is a significant problem, as the severity of this condition informs the actions to be taken by the healthcare team [1]. In everyday practice, therapies are guided using instruments that rely primarily on hierarchical ratings to categorize the severity of respiratory problems through scales or scores based on biometric characteristics [2]. Therefore, to establish whether a hierarchical system is consistent, it must be subjected to a process of analysis of its psychometric properties, particularly its reliability [3].

Scales have been developed through consensus, protocols, or conveniences that respond to real clinical problems. Applying a methodological and scientific basis to any instrument is essential to evaluate its performance. Using a tool without having subjected it to a control, validation, or comparison system compromises its reliability. Therefore, these tools must be supported by analyses derived from these biometric processes [2,3]. In this context, there is a legitimate issue over the actual impact of scales in physiotherapy; however, their usage is increasing. Most of these scores consist of assigning values to a set of variables that categorize the severity of a disorder, which, in this particular case, is to assess respiratory compromise [4].

In this context, the Ventilatory Workload Kinetic Index (VWKI) has been proposed as a clinical instrument to evaluate ventilatory balance–imbalance and standardize the assessment of the respiratory system [5] based on a theoretical model that recapitulates the imbalance between ventilatory needs and neuro-cardio-respiratory capacities [6]. In such a condition, it is important to determine whether instruments like scales can effectively evaluate the complexity of the symptomatology and clinical status in various instances [7]. Despite technical advancements that have contributed to minimizing outcome variability, dependable clinical measurement in its “bedside” application remains a learning process.

Specifically, the VWKI is a clinical instrument proposed by Escobar et al. (2000) to categorize the respiratory compromise of hospitalized patients to optimize physiotherapy resources. It consists of eight variables, each with a score ranging from zero or “normal” to three points, which is the greatest severity. The sum of the eight variables generates the total score of the VWKI. Complementary to this, the variables are classified into loads, internal or external biophysical phenomena that increase the mechanical or physiological output of the ventilatory system; translations, a set of variables that enables adequate visualization and monitoring of the respiratory symptoms; and supports, internal or external biophysical adjustments that contribute to “stabilizing” the respiratory symptoms [4,6].

In addition to providing professional care and staying up to date with their knowledge through continuous training, one of the respiratory specialist’s tasks is to establish competencies demonstrating the reliability and importance of their clinical measurements. This allows them to effectively control and use specialized instruments in their field of expertise [7]. Thus, the regulated application of scales to comprehensively assess the object of study and the reliability of such scales should be a continuous, systematic, and situated process [8]. Therefore, this study sought to determine the reliability of the VWKI between two evaluators and its applicability in clinical physiotherapy.

## 2. Materials and Methods

This cross-sectional exploratory study was conducted in January 2024 at the Hospital El Carmen de Maipú (HEC). The following services were considered: medicine, intensive care unit (ICU), and others (Surgery and Geriatrics). This study was approved by the Scientific Ethics Committee (letter N° 39/2024) of the Central Metropolitan Health Service. The patients or their legal guardians provided written informed consent to participate in the study.

### 2.1. Study Design

**Figure 1:**
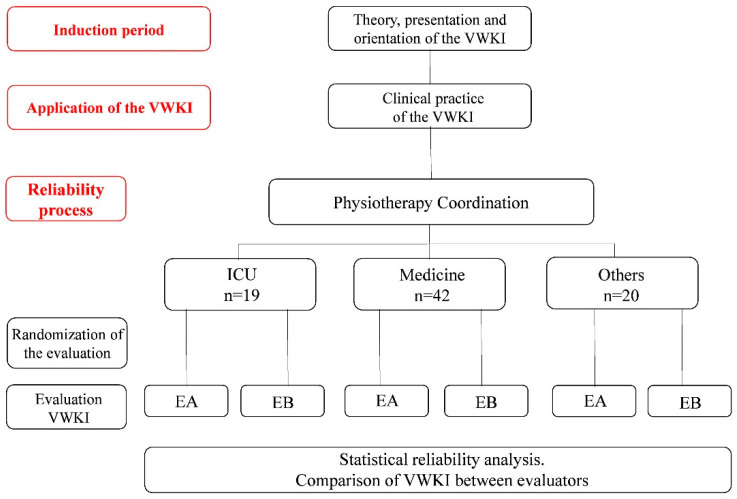
The three phases of the study are highlighted in red.

The study was divided into three stages: (i) induction period, (ii) application of the VWKI, and (iii) reliability process (Figure 1). In parallel to the reliability process, the following information was retrieved from each participant’s file on the assessment day: (i) medical diagnosis, treatment, history of co-morbidities, and days of hospitalization. Finally, the physiotherapy coordinator reported the prioritization of each participant’s care, which were priority 1 (≥2 visits) and priority 2 (1 visit).

#### 2.1.1. Induction Period

To optimize the clinometry of the scale, an induction period was developed, which was divided into two stages: (i) presentation and orientation: 2 expository sessions of one hour each were held, where the VWKI was presented, and the material was distributed for discussion; (ii) feedback: 2 sessions of one hour each where questions were answered, and behavior was standardized for the evaluation of the different variables of the VWKI.

#### 2.1.2. Application of the VWKI

The implementation of the VWKI in clinical practice consisted of evaluating 10 patients guided by the expert. It should be noted that the construction of the scale used is given under a theoretical model of loads and supports according to Vassilakoupoulos [6]. The VWKI was measured as follows: (i) additional oxygen contribution (O_2_) and oxygen saturation (SO_2_), (ii) respiratory rate (RR), (iii) use of inspiratory and/or expiratory accessory musculature (UAM), (iv) pulmonary murmur (PM) and airway resistance (AR), (v) cough and attempts to permeabilize the airway (APA).

##### Respiratory Rate (RR)

The number of breaths in one minute was recorded. It was measured with a Casio stopwatch (model Hs-3v-1b). The data were scored according to Table 1.

##### Additional Oxygen Contribution (O_2_)

The additional oxygen support administered was measured as a programmed percentage regardless of the system used (high or low flow). The data were scored according to Table 1.

##### Oxygen Saturation (SO_2_)

This was recorded with a NONIN pulse oximeter (ONYX 9500, Plymouth, MN, USA) attached to the patient’s index finger. The data were scored according to Table 1.

##### Use of Accessory Muscles (UAM)

Accessory muscle activity was measured by observation and/or contact in a semi-seated position (45°) with minimal intervention to clearly measure the actual involvement of the accessory muscles. The data were scored according to Table 1.

##### Pulmonary Murmur (PM)

This was measured with a 3M™ Littmann^®^ Classic III stethoscope (Saint Paul, MN, USA). The central points of each of the ten quadrants were estimated in total lung capacity. Specifically, frontal quadrants were located at the two apices, lateral quadrants were located at the two bases, and posterior quadrants were identified at the two highest points, two middle points, and two lowest points. Each location received points: 0 points for restricted pulmonary murmur, 1 point for decreased pulmonary murmur, and 2 points for suppressed pulmonary murmur. The sum of the ten locations was classified according to Table 1.

##### Airway Resistance (AR)

Once the inspiratory and expiratory phases were defined, the presence or absence of prolonged expiration, expiratory wheezing or biphasic wheezing was determined. A 3M™ Littmann^®^ Classic III stethoscope (Saint Paul, MN, USA) was used. The data were scored according to Table 1.

##### Cough (Cough)

Its evaluation was clinical and determined by the kinematic observation of a voluntary coughing effort: (i) normal presence of the three phases, (ii) alteration of the preparation stage (inspiratory reserve volume), and (iii) alteration of the compressive expulsion phase or coughing mechanism absent. The data were scored according to Table 1.

##### Attempts to Permeabilize the Airway (APA)

The score related to the number of attempts needed to eliminate the mucous component through coughing was determined according to Table 1.

#### 2.1.3. Reliability Process

The required sample was calculated using the software G*Power v. 3.1.9.7 with a power of 80%, a significance level of 0.05, and an effect size (f) of 0.4 based on the reduction in the VWKI reported by Muñoz-Cofré et al. (2024) [1]. According to the sample size calculation, a minimum of 81 measurements was required to detect a difference in VWKI. This must be an a priori calculation of a measure in the hospitalized population; a dropout percentage was not considered. Evaluator A (EA) was a physiotherapist from the Physical Medicine and Rehabilitation Service of the HEC, and Evaluator B (EB) was a physiotherapist expert in respiratory assessment (Chilean Ministry of Health) with 20 years of experience. The participants were randomly selected on the day of the assessment, and the application of the VWKI was isotemporal, so the variables were not modified. After applying the VWKI, the EA and EB recorded the values of each variable separately to avoid bias. Inclusion criteria were (i) 18 years of age or older, (ii) hospitalized in medicine, ICU, surgery, or geriatrics, (iii) oriented in time and space and able to cooperate in the respiratory assessment. Exclusion criteria were (i) being connected to non-invasive mechanical ventilation, (ii) being connected to invasive mechanical ventilation, (iii) weaning patients on a tracheal cannula, (iv) patients with an indication not to mobilize, and (v) patients discharged from the hospital on the day of the assessment.

### 2.2. Statistical Analysis

The SPSS software version 25.0 was used for all analyses. Median, minimum, and maximum scores were calculated for each variable and the total score of the VWKI. Inter-rater reliability (EA and EB measurements) was analyzed using the intraclass correlation coefficient (ICC) with a 95% confidence interval (CI). The following interpretation of ICC was used: <0.5, poor reliability; 0.51 to 0.75, moderate reliability; 0.76 to 0.90, good reliability; and >0.90, excellent reliability. For comparisons of VWKI by user prioritization and by services, the normality of the data was first determined using the Kolmogorov–Smirnov test. Then, Student’s *t*-test or the Mann–Whitney U test and an ANOVA or Kruskal–Wallis test, respectively, were used. The level of significance was set at *p* < 0.05.

## 3. Results

Concerning the stability of the VWKI variables (Table 2), loads such as PM obtained good inter-rater reliability (ICC = 0.770, *p* < 0.05). In translations, RR and APA showed good inter-rater reliability (ICC = 0.831, *p* < 0.05; ICC = 0.889, *p* < 0.05, respectively), and the SO_2_ obtained excellent inter-rater reliability (ICC = 0.986, *p* < 0.05), while in supports, O_2_ showed excellent reliability (ICC = 0.982, *p* < 0.05) and cough showed good reliability (ICC = 0.820, *p* < 0.05). Finally, the VWKI total score obtained excellent inter-rater reliability (ICC = 0.913, *p* < 0.05) (Table 2). On the other hand, AR showed moderate inter-rater reliability (ICC = 0.528, *p* < 0.05), while UAM showed poor inter-rater reliability (ICC = 0.483, *p* < 0.05) (Table 2).

**Figure 2:**
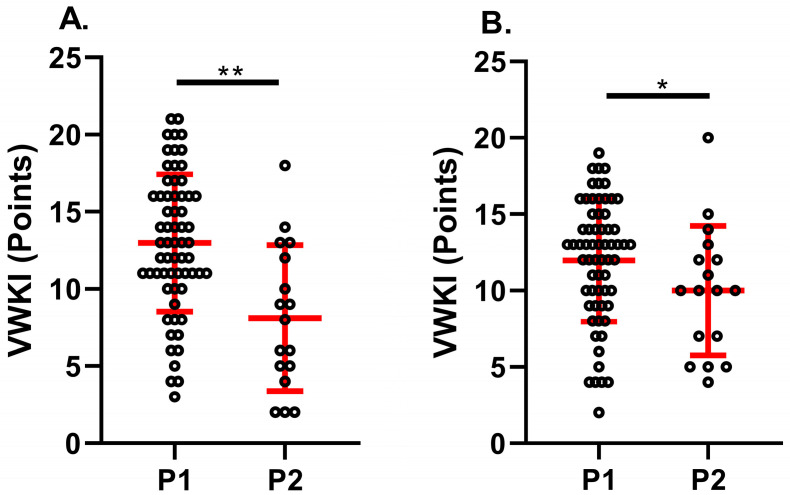
Comparison of VWKI according to the prioritization system in the HEC. (**A**). Evaluator A; (**B**). Evaluator B. P1: priority 1; P2: priority 2; **: *p* = 0.01; *: *p* < 0.05. Student’s statistical *t*-test.

**Figure 3:**
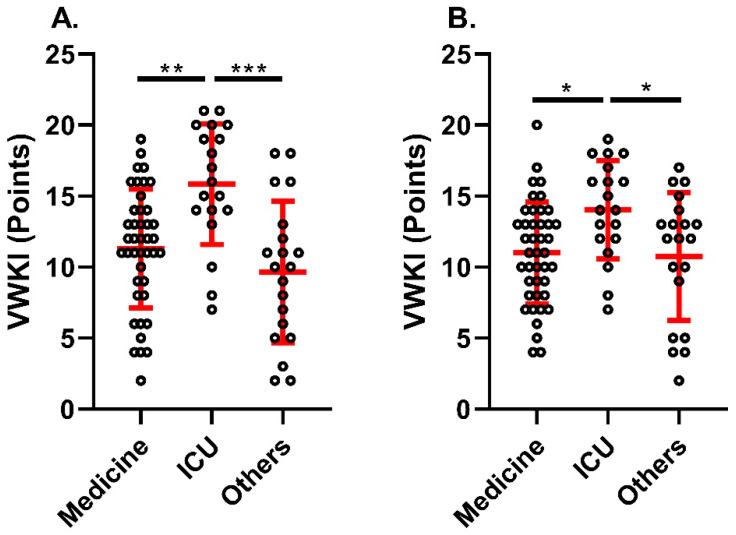
VWKI according to service. (**A**). Evaluator A; (**B**). Evaluator B. ***: *p* = 0.0001; **: *p* = 0.01; *: *p* < 0.05. Kruskal–Wallis statistical test.

With respect to the physiotherapy care prioritization system, for both EA (P1 = 13 [3–21]; P2 = 8 [2–18] points) and EB (P1 = 13 [2–19]; P2 = 10 [4–20] points), the total VWKI score was significantly higher in priority 1 (*p* = 0.001; *p* < 0.05, respectively) (Figure 2). However, when comparing the total VWKI score by services, both EA and EB rated the VWKI significantly higher in the ICU than in medicine and the other services (*p* = 0.001, *p* = 0.0001 and *p* < 0.05, *p* < 0.05, respectively) (Figure 3).

## 4. Discussion

This study aimed to determine the inter-rater reliability and applicability of the VWKI in the clinical physiotherapy setting. The main results are as follows. (i) The VWKI total score had excellent inter-rater reliability. (ii) The AR and UAM variables had low reliability. (iii) The VWKI was consistent with the physiotherapeutic care prioritization system, i.e., the VWKI was significantly higher in priority 1 patients. (iv) The VWKI was able to determine the complexity of the patients; thus, the score of the patients in the ICU was significantly higher than those in the other units. In terms of clinical intervention, both the stability of the data obtained by the assessment tool and the discrimination of the data could contribute to the prioritization of intervention and clinical decision making [3]. Therefore, we believe it is important to highlight that using the VWKI is not conditioned to a specific health care team member. The findings of this study indicated a high level of reliability in the overall VWKI score. Consequently, the application of this instrument should be contingent upon a thorough acquisition process followed by the validation of its use and interpretation. The application of the VWKI would serve a dual purpose. As in an initial evaluation, it could determine the characteristics of the respiratory symptoms and would then contribute to the detailed monitoring of the behavior of the variables in patients.

One of the main findings of this study was the excellent inter-rater reliability of the VWKI total score (ICC = 0.913, *p* < 0.05). This agrees with Cabib et al. (2004), who assessed the inter-rater reliability of the VWKI between two physiotherapists in patients ventilated with pressure support (PVPS). Their results showed the same median VWKI for both physiotherapists (EA= 11 (6–18), EB= 11 (6–17); *p* = 0.9, K = 0.84). In conclusion, the VWKI applied in PVPS is a reliable inter-physiotherapist instrument [9]. It is important to note that the sample of the present study (i) did not include PVPS, and (ii) the sample was recruited from three services with different complexity. Therefore, the reliability of the VWKI would be independent of the patient’s condition and the service in which they are hospitalized. In this way, having a source of intra-hospital information with discriminatory characteristics from the hierarchy of commitments allows for the planning of human and material resources, which, in contexts of high demand, favors adequate decision making associated with a standardized process [7].

An important point to consider is the low reliability obtained in the AR (0.528 < 0.05) and UAM (0.483 < 0.05) variables, the evaluative elements of which are more subjective in the evaluators’ assessment. This has been reported as one of the elements that should be handled with greater care given the clinical implications that derive from these variables, namely the balance between the muscular contraction needed to break the lung–thorax elastance [6]. In this regard, the experts recommend emphasizing the standardization of measurement methods, observer training, and measurement automation [10]. Therefore, it would be very useful to generate complementary material, in addition to the scoring table, that explains and clarifies point by point the situations that could generate false positives in the VWKI measurement.

The VWKI was consistent with the physiotherapy care prioritization system proposed by the HEC physiotherapy coordination; here, patients with two or more attendances had a significantly higher total VWKI score than patients with one attendance. This is consistent with the findings of Quintero et al. (2016), who aimed to explain, automate, and disseminate the usefulness of the VWKI. They concluded that the systematization of the VWKI would allow them to stratify the risk of pulmonary failure of hospitalized patients and to address, objectively and specifically, the respiratory physiotherapeutic treatment of each patient according to their characteristics [11]. In clinical practice, the ability to prioritize care in a service and/or a group of assigned patients is a value associated with the orientation of technical efforts in terms of procedural urgency, which does not necessarily coincide with biomedical urgency. The categorization of respiratory impairment using the total VWKI score could optimize treatment interventions, taking into account the severity and specific aspects of the respiratory disorder assessed [3].

Finally, to emphasize the “sensitivity” of the VWKI in assessing the physiotherapy complexity of patients, it is important to note that while the ICU score was significantly higher than the Medicine Service and other units, this difference is not directly related to the amount of time or the level of difficulty involved in the procedures. In 2004, a study was conducted to examine the behavior of the VWKI in patients with varying degrees of medical complexity. The findings revealed a substantial correlation between higher levels of attendance and an increase in VWKI (VWKI 17 (11–23) vs. 7 (4–14); *p* < 0.0001), which is a confounding factor when medical complexity is assimilated with kinesthetic severity [12]. In this regard, Muñoz et al., 2024 concluded that the VWKI serves to identify ventilatory problems in outpatients and can also distinguish where the greatest deterioration lies, considering the differentiated analysis of loads, translations, and supports, which does not necessarily correspond to the levels of intervention, which are often stereotyped in respiratory physiotherapy [10,12].

Certain limitations in this study must be acknowledged. (i) The imbalance in the number of patients per service is due to the HEC’s clinical reality, which necessitates adjusting the number of beds based on the epidemiological behavior of the in-patient population. This ethical consideration makes it impossible to choose patients selectively. (ii) The duration of the theoretical training of the VWKI should be reassessed in light of the experience gained. (iii) The evaluations were reduced to only three services, so it would be necessary to establish the reality of the ventilatory compromise of the entire hospital center.

## 5. Conclusions

The conclusions drawn in this study were the excellent reliability of the total score of the VWKI, its consistency with the physiotherapy care prioritization system, and the ability to determine the severity of clinical respiratory symptoms.

## Figures and Tables

**Table 1:** Division and score of the variables of the Ventilatory Workload Kinetic Index.

RR	O_2_ (%)	SO_2_ (%)	UAM	PM	AR	Cough	APA	SCORE
10–16	100–98	21	Without UAM	0	Without AR	Spontaneous or effective cough	Not required	0
17–25	97–95	22–28	Diaphragmatic overload	1–7	Prolonged expiration	Threshold disorder or inspiratory reserve volume ↓	2 attempts	1
26–34	94–92	29–49	AMR I or E	8–14	Wheezing or expiratory rhonchi	Compressive or expulsive phase altered	3–4 attempts	2
35+	<91	>50	AMR I and E/PR	15–20	Wheezing or expiratory and inspiratory rhonchi	Absent or severely altered mechanism	>5 attempts	3

RR: respiratory rate; O_2_: additional oxygen contribution; SO_2_: oxygen saturation; UAM: use of accessory muscles; PM: pulmonary murmur; AR: airway resistance; APA: attempts to permeabilize the airway; AMR: accessory muscle recruitment; I: inspiratory; E: expiratory; PR: paradoxical respiration. Modified from Escobar et al. (2000) [4].

**Table 2:** Reliability of the VWKI variables and the total score.

	Evaluator A	Evaluator B	ICC (CI 95%)	*p* Value
Loads				
PM	2 (0–3)	2 (0–3)	0.770 (0.538–0.819)	<0.05
AR	2 (0–3)	1 (0–3)	0.528 (0.247–0.702)	<0.05
Translations				
RR	1 (0–2)	1 (0–3)	0.831 (0.797–0.891)	<0.05
SO_2_	2 (0–3)	2 (0–3)	0.986 (0.978–0.991)	<0.05
UAM	1(0–3)	2 (0–3)	0.483 (-0.043–0.724)	<0.05
APA	1 (0–3)	1 (0–3)	0.889 (0.828–0.928)	<0.05
Supports				
O_2_	1 (0–3)	1 (0–3)	0.982 (0.972–0.988)	<0.05
Cough	2 (0–3)	2 (0–3)	0.820 (0.720–0.884)	<0.05
VWKI Total	11 (2–21)	12 (2–20)	0.913 (0.864–0.944)	<0.05

The values are presented as median (minimum–maximum). ICC: intraclass correlation coefficient; CI: 95% confidence interval; RR: respiratory rate; O_2_: additional oxygen contribution; SO_2_: oxygen saturation; UAM: use of accessory muscles; PM: pulmonary murmur; AR: airway resistance; APA: attempts to permeabilize the airway; VWKI: Ventilatory Workload Kinetic Index.

## Data Availability

The data used in this research is available. Please send request to Dr. Rodrigo Muñoz-Cofré.
